# Digital PET/CT allows for shorter acquisition protocols or reduced radiopharmaceutical dose in [^18^F]-FDG PET/CT

**DOI:** 10.1007/s12149-021-01588-6

**Published:** 2021-02-07

**Authors:** Ian Alberts, Christos Sachpekidis, George Prenosil, Marco Viscione, Karl Peter Bohn, Clemens Mingels, Kuangyu Shi, Ali Ashar-Oromieh, Axel Rominger

**Affiliations:** Department of Nuclear Medicine. Inselspital, Bern University Hospital, University of Bern, Freiburgstr. 18, 3010 Bern, Switzerland

**Keywords:** List mode acquisition, PET/CT, Positron emission tomography, Digital PET

## Abstract

**Purpose:**

To establish the feasibility of shorter acquisition times (and by analogy, applied activity) on tumour detection and lesion contrast in digital PET/CT.

**Methods:**

Twenty-one randomly selected patients who underwent oncological [^18^F]-FDG PET/CT on a digital PET/CT were retrospectively evaluated. Scan data were anonymously obtained and reconstructed in list-mode acquisition for a standard 2 min/bed position (bp), 1 min/bp and 30 s/bp (100%, 50% and 25% time or applied activity, respectively). Scans were randomized and read by two nuclear medicine physicians in a consensus read. Readers were blind to clinical details. Scans were evaluated for the number of pathological lesions detected. Measured uptake for lesions was evaluated by maximum and mean standardized uptake value (SUVmax and SUVmean, respectively) and tumour-to-backround ratio (TBR) were compared. Agreement between the three acquisitions was compared by Krippendorf’s alpha.

**Results:**

Overall *n* = 100 lesions were identified in the 2 min and 1 min/bp acquisitions and *n* = 98 lesions in the 30 s/bp acquisitions. Agreement between the three acquisitions with respect to lesion number and tumour-to-background ratio showed almost perfect agreement (K’s *α* = 0.999). SUVmax, SUVmean and TBR likewise showed > 98% agreement, with longer acquisitions being associated with slightly higher mean TBR (2 min/bp 7.94 ± 4.41 versus 30 s/bp 7.84 ± 4.22, *p* < 0.05).

**Conclusion:**

Shorter acquisition times have traditionally been associated with reduced lesion detectability or the requirement for larger amounts of radiotracer activity. These data confirm that this is not the case for new-generation digital PET scanners, where the known higher sensitivity results in clinically adequate images for shorter acquisitions. Only a small variation in the semi-quantitative parameters SUVmax, SUVmean and TBR was seen, confirming that either reduction of acquisition time or (by analogy) applied activity can be reduced as much as 75% in digital PET/CT without apparent clinical detriment.

## Introduction

The recent introduction of digital PET/CT scanners for routine clinical use represents a significant milestone for nuclear medicine and molecular imaging. Although PET/CT scanners have always been truly “digital” insofar as their outputs were in the form of digital signals, the replacement of analogue photomultiplier tubes with solid-state detection systems resulted in the first fully “digital” PET/CT, and has surmounted many of the inherent physical limits placed by previous-generation analogue technologies. These new fully digital systems exhibit a plethora of technical advantages, which include a better coupling between the crystal and photodetectors, improved background-to-noise, faster time-of-flight (TOF) and associated advanced TOF reconstruction. In addition, state-of-the-art digital systems often include longer axial coverage, smaller crystals, and more advanced electronics, which lead to higher sensitivity, higher spatial resolution, and shorter deadtime. These improved performance characteristics have been confirmed by a number of publications [1, 2], which correspond to improvements in image quality and lesion detection [2–6].

Whereas the current iteration of the European Asociation of Nuclear Medicine (EANM) guidelines report examination protocols for analogue scanners and for “step-and-shoot” type acquisitions [7], no current guidelines are yet available for systems with digital acquisition. Although the performance characteristics, and EANM Research Ltd (EARL) compliance of such scanners have been reported [8–10], the full clinical potential of digital scanners is yet to be fully characterized. In particular, whereas the manufacturers’ literature report shorter-acquisition times and/or reduced activity as potential improvements of such systems, the advantage for the patient needs to be investigated and verified in a clinical setting.

The aim of this present study is to assess the effect of short acquisition times or reduced activity on lesion detectability as assessed by two nuclear medicine physicians, followed by lesion uptake quantitation in a digital scanner.

## Materials and methods

### Patient population

In this retrospective analysis we included 21 randomly selected individuals who were examined on our digital PET/CT (dPET/CT) at the University Hospital Bern, Inselspital and whose PET datasets were available for anonymous analysis. All patients underwent a clinically routine [^18^F]-FDG PET/CT for oncological purposes. Patient characteristics are outlined in Table 1.

**Table. 1:** Number of lesions detected by dPET/CT at 2 min/bed position (bp), 1 min/bp and 30 s/bp list-mode acquisition and patient characteristics

Patient	Number of pathological lesions				Patient characteristics			BMI
	30s/bp	1min/bp	2min/bp		Dose (MBq)	mSV	Weight	
1	1	1	1		217	4.1	63	21.0
2	13	13	13		234	4.4	64	21.6
3	9	10	10		234	4.4	69	20.3
4	1	1	1		304	5.7	86	25.9
5	0	0	0		132	2.5	38	15.6
6	1	1	1		385	7.3	100	27.7
7	1	1	1		166	3.1	46	17.5
8	3	3	3		333	6.3	99	28.9
9	3	3	3		175	3.3	48	17.6
10	0	0	0		215	4.0	60	20.5
11	1	1	1		338	6.4	98	31.6
12	20	20	20		321	6.0	92	35.0
13	10	11	11		259	4.9	73	26.8
14	1	1	1		230	4.3	62	23.3
15	20	20	20		342	6.4	100	30.8
16	7	7	7		219	4.1	55	21.4
17	0	0	0		266	5.0	75	23.1
18	1	1	1		312	5.9	89	25.7
19	1	1	1		308	5.8	86	28.7
20	4	4	4		167	3.1	46	18.9
21	1	1	1		256	4.8	75	24.2
Total	98	100	100	Mean	257.7619	4.8	72.57143	24.1

*Dose* applied dose in MBq, effective dose for an adults in *mSV* effective dose for an adult (1.9 × 10^−2^ mSv/MBq [7]), weight (kg), BMI (kg/m^2^)

### Imaging protocol

Patients were required to fast for > 6 h prior to scanning and finger-prick blood glucose measurement prior to scanning confirmed a venous blood glucose of < 120 mg/dl. 3.5 MBq/kg of [^18^F]-FDG was applied intravenously as per clinical routine. Scans were acquired at 60 min post injection of radiotracer (p.i.). All patients received regular whole-body PET scans (from skull base to the thighs) on a Biograph-Vision 600 Edge PET/CT digital scanner (Siemens, Erlangen, Germany). A non-contrast-enhanced CT scan was performed 1 h post tracer injection with slice thickness of 1.0 mm, pitch factor 1, bone and soft tissue reconstruction kernels and maximum of 120 kV and 90 mAs by applying CARE kV and CARE Dose. Immediately after CT scanning, a whole-body PET (skull base to thighs) was acquired in 3D (matrix: 440 × 440) with a zoom factor of 1.0. The emission data were corrected for randoms, scatter and decay. Reconstruction was conducted with TrueX (Point Spread Function, PSF) + time-of-flight (TOF) algorithm and 2 mm Gauss-filter was applied. TrueX is an iterative resolution recovery algorithm with PSF modelling included. Attenuation correction was performed using the low dose non-enhanced computed tomography data. Images were obtained in list mode. PET data were reprocessed to produce sinograms corresponding to 2 min/bed position (bp), 1 min/bp, and 30 s/bp.

### Image evaluation

Image analysis was performed using an appropriate workstation and software (SyngoVia; Siemens, Erlangen, Germany). Two experienced physicians (one board certified nuclear medicine physician and one experienced resident, both with experience in reading digital PET/CT scans) read all scans together. Disagreements were resolved by consensus. Readers were blinded to patient demographics and clinical details when reviewing scans. Neither reader had previously read or had familiarity with any of the included patients. The order of the scans was randomized prior to each read. Starting first with the reconstructions assumed to deliver the lowest quality, we analysed the 30 s/bp acquisitions with respect to number of pathological lesions. After a 48 h waiting period, the scans were then re-randomized and the 1 min/bp, and likewise the 2 min/bp scans analysed. The number of lesions judged to be pathological were counted, using previously published interpretation criteria [7]. To avoid discrepancies in the counting of polymetastatic individuals, a maximum of 20 lesions were counted per individual. Insofar as possible, recall bias was limited by reading each set of reconstructions starting first with the presumed lowest quality, with scans in randomized order, anonymized to patient demographics and with a 48 h waiting period between each reading session.

Scans were then re-analysed by both readers, this time comparing all three reconstructions with respect to lesion radiotracer uptake concomittantly. In patients with multiple lesions, up to five of the most visually prominent lesions in terms of radiotracer uptake were analysed to prevent overrepresentation by polymetastastic individuals. Lesion uptake was calculated by placing a volume-of-interest (VOI) around the lesion with 40% isocontour as previously published [11] and SUVmax and SUVmean were recorded. Background uptake was measured by placing a 14cm^3^ volume-of-interest in normal liver tissue in the right liver lobe as previously described [12]. Tumour-to-background ratio (TBR) was defined as SUVmax (lesion)/UVmean (background) and taken as a measurement of lesion contrast and visibility. Regions of interest were copied and pasted between different list-mode acquisitions, ensuring that the same volume-of-interest was analysed for each acquisition.

### Statistical analysis

Statistical analyses were performed using Excel (Microsoft, Redmond, Washington) and Graphpad Prism Version 6 (San Diego, California).

Agreement between the three acquisitions for number of lesions detected and for TBR were compared by Krippendorf’s *α*. Paired differences between acquisitions for lesion TBR, SUVmax and SUVmean were compaired by the paired student’s *t*-test with correction for a log-normal distribution and Bonferonni correction, *p* values < 0.05 were considered statistically significant.

### Sample size estimate

The null hypothesis is that the correlation in terms of lesion detection between the 2 min/bp and 30 s/bp scans is < 0.65, the alternative hypothesis is that the correlation is > 0.65. For a two sided $\alpha$ = 0.05 and *β* = 0.1, a sample of *n* = 20 was calculated. *N* = 21 patients were included. Inclusion criteria were patients undergoing a routine oncological PET/CT in our department. Exclusion criteria were aborted scans, non-adherence to fasting requirements or initial blood glucose > 120 mg/dl.

## Results

### Lesion detection rate

A total of *n* = 100 malignant lesions were counted in *n* = 18 individuals, with two polymetastatic individuals (*n* > 20 lesions, with a maximum of 20 lesions analysed). Negative scans were observed for *n* = 3 indviduals. Of the *n* = 100 lesions, 100 were detected at 2 min/bp and 1 min/bp acquisition, and 98 at 30 s/bp; these two lesions missed in the 30 s/bp acquisitions were in polymetastatic individuals and would not have influenced the patient’s staging or further management. In terms of lesion detection, the correlation between the 30 s/bp and 2 min/bp was therefore 98%, confirming the alternative hypothesis. The results are shown in Table 1.

### Lesion detectability

**Fig. 1:**
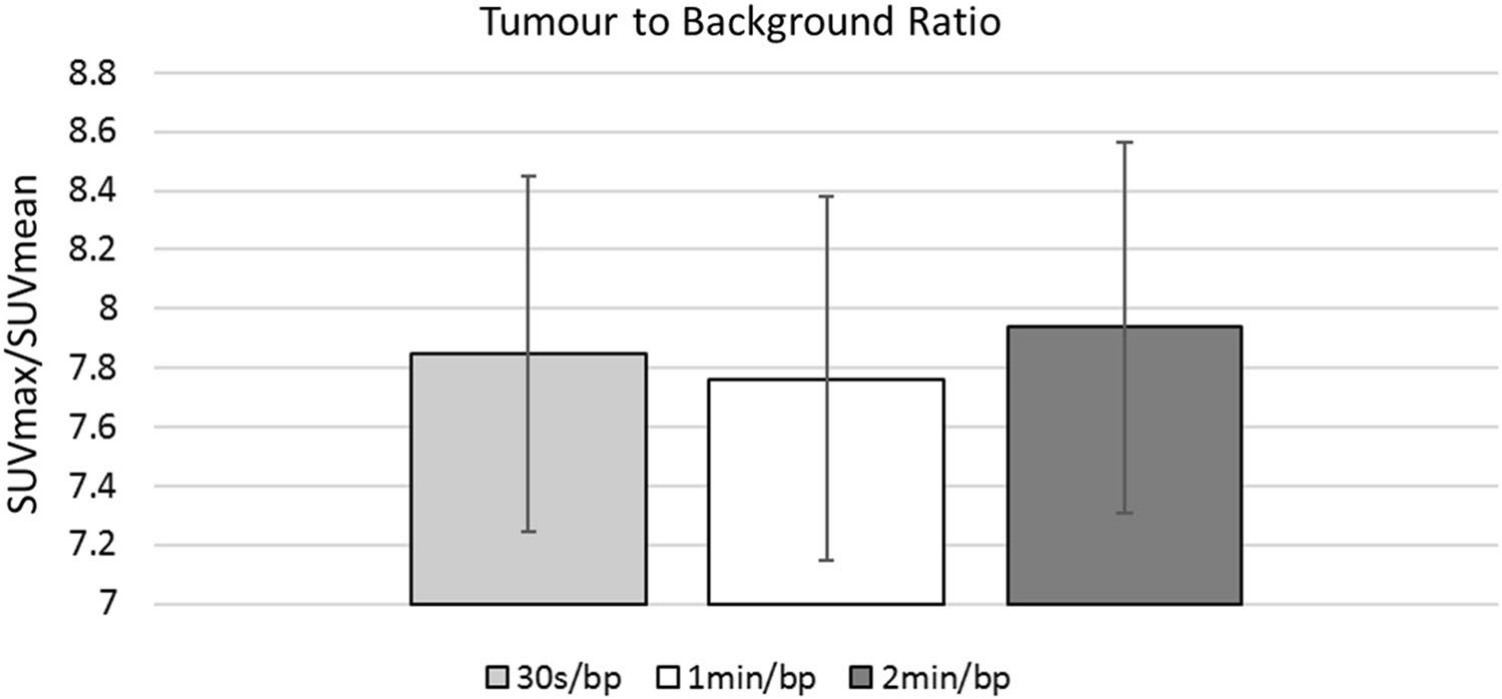
Tumour-to-background ratio for the three acquisitions [30 s/bed position (bp); 1 min/bp; 2 min/bp]. The bars represent the standard error in the mean. No clear relationship between list-mode acquisition length and TBR was observed

Lesion detectability was compared semi-quantiatively for the three acquisition modes by calculation of the tumour-to-background ratio (TBR) as described above for n = 49 lesions. The results are shown in Fig. 1. Slightly higher mean TBR was seen at 2 min/bp compared to 1 min/bp (7.94 vs 7.76, *p* = 0.02) with no difference between 1 min/bp and 30 s acquisitions (7.76 vs 7.84, *p* = 0.29). Overall, agreement between the three acquisitions was high (K’s alpha = 0.99). Mean variability between the 30 s and 2 min/bp acquisitions was 0.79%.

### Lesion uptake

Lesion SUVmax and SUVmean were considered between the three acquistions, and is shown in Table 2. Slightly higher SUVmean was observed for the 2 min/bp acquisitions compared to 30 s/bp (*p* = 0.0001). In Fig. 4 we show SUVmax at different time points and mean SUVmax. Paired student’s *t*-test with Bonferonni correction reveal no signfificant differences between 30 s and 2 min, *p* > 0.05 for TBR. Likewise, no significant differences are seen for SUVmax (30 vs. 2 min; *p* > 0.05). Mean variability between the 30 s and 2 min/bp acquisitions was 0.35% for SUVmean and 0.77% for SUVmax.

**Table 2:** Measured lesion SUVmax and lesion SUVmean (± standard deviation, SD) for the three acquisitions

	Acquisition	Mean	SD
SUV max	30 s	15.11	9.38
(Lesion)	1 min	14.62	9.30
	2 min	14.99	8.73

SUV mean	30 s	8.57	5.38
(Lesion)	1 min	8.51	5.46
	2 min	8.73	5.60

SUV mean	30 s	2.05	0.27
(Background)	1 min	2.02	0.28
	2 min	2.02	0.29

## Discussion

**Fig. 2:**
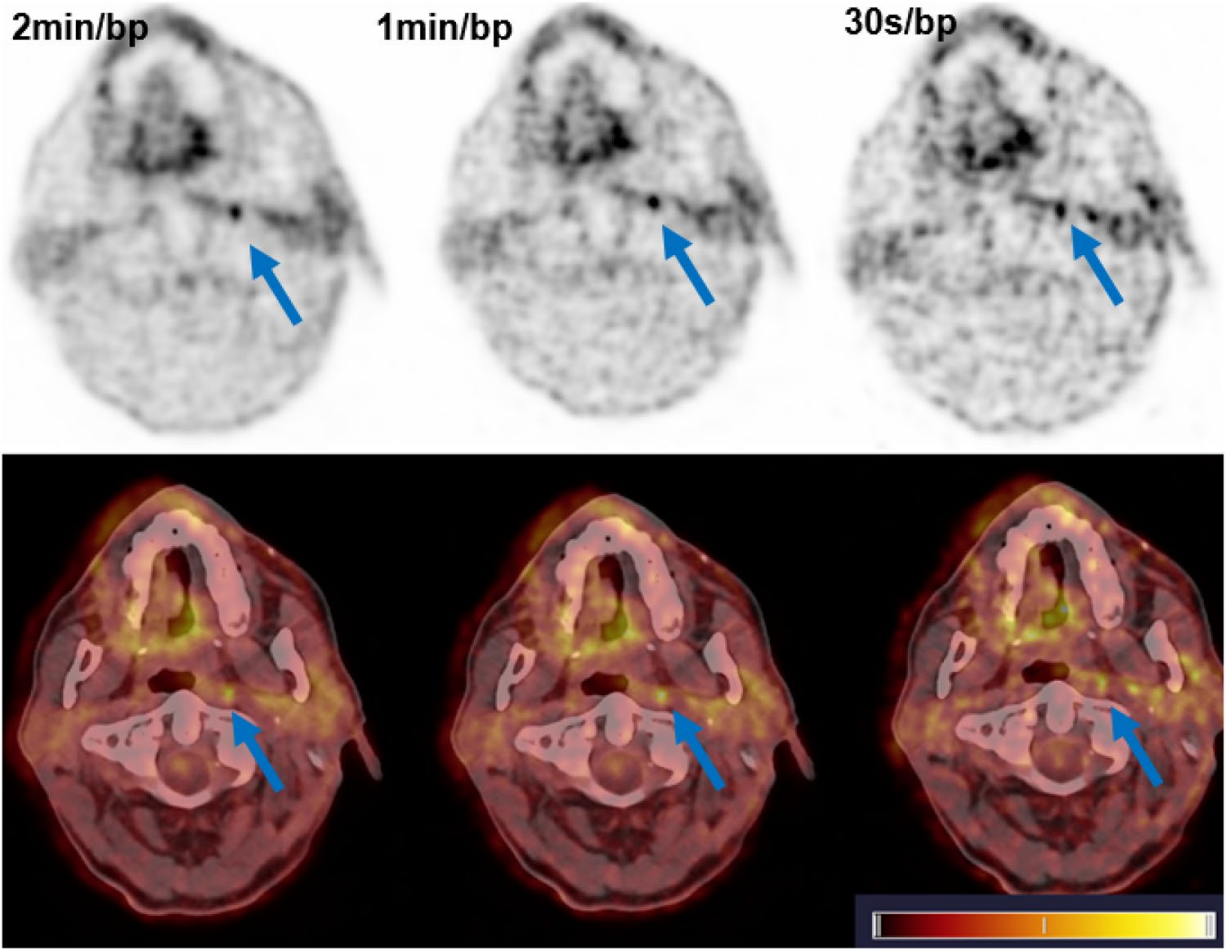
Example of lesion missed at shorter acquisition (30 s/bp) but easily identified at 1 min and 2 min/bp list-mode acquisition. This retropharyngeal lymph node (blue arrow) was overlooked owing to increased noise in the 30 s/bp acquisitions, and is much better delineated at 2 min and 1 min/bp. The top row represents the PET data, the bottom row fusion of PET and CT. Bottom right shows colour look-up table (scale 0–8 SUV)

In *n* = 21 patients undergoing routine oncological [^18^F]-FDG PET/CT on a digital scanner, we find that 98% of lesions (*n* = 98/100) are detected at a table velocity equivalent to 30 s/bp (25% of standard acquisition time). For the two “missed” lesions, we note that these were in polymetastatic individuals where the extra lesions could be slightly better discriminated, the extra lesion however would not have influenced the patient’s stage. Example images for one such patient is given in Fig. 2.

**Fig. 3:**
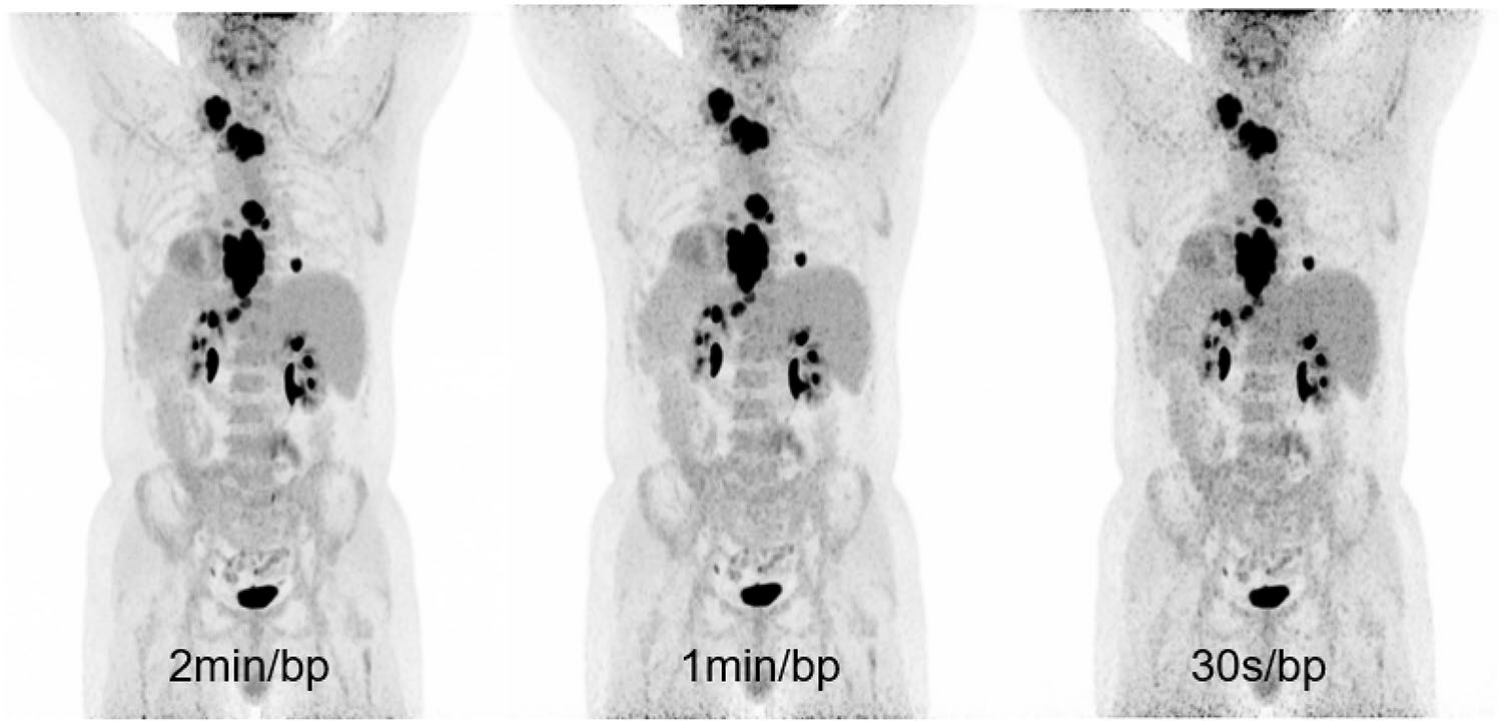
Example of increased noise for the 30 s/bp acquisitions in the maximum intensity projection images (MIP), left most image 2 min/bp, middle 1 min/bp, rightmost 30 s/bp. Note that the increased noise, while subjectively reducing image quality, does not influence the detectability of mediastinal and cervical lymph node lesions in this patient

Only minimal variations in TBR and SUVmax and SUVmean were observed between the three list-mode acquisitions, with almost perfect agreement (Krippendorf’s *α* = 0.99) between them, suggesting that shorter acquisitions are adequate for routine clinical purposes. We find the shorter acquisitions to be of slightly lower subjective image quality, although such subjective impressions were not the focus of this present study, but rather the aim was to quantify the magnitude of any clinical effect such shorter-acquisition protocols may have. While such acquisitions may be less aesthetically pleasing, we find no evidence of clinical detriment. We contend that while a modestly higher background noise was observed in the 30 s/bp images (as exemplified by Fig. 3), this was not associated with any objective disadvantage.

Given the known relationship between applied radiopharmaceutical dose, acquisition time and count statistics, by analogy, our data can also be interpreted as supportive of a reduction in applied activity in dPET/CT. Indeed, with a mean effective dose (excluding the CT component) of 4.9 mSv (Table 1), the 30 s/bp reconstructions are equivalent to a 75% reduction in dose, or 3.7 mSv.

**Fig. 4:**
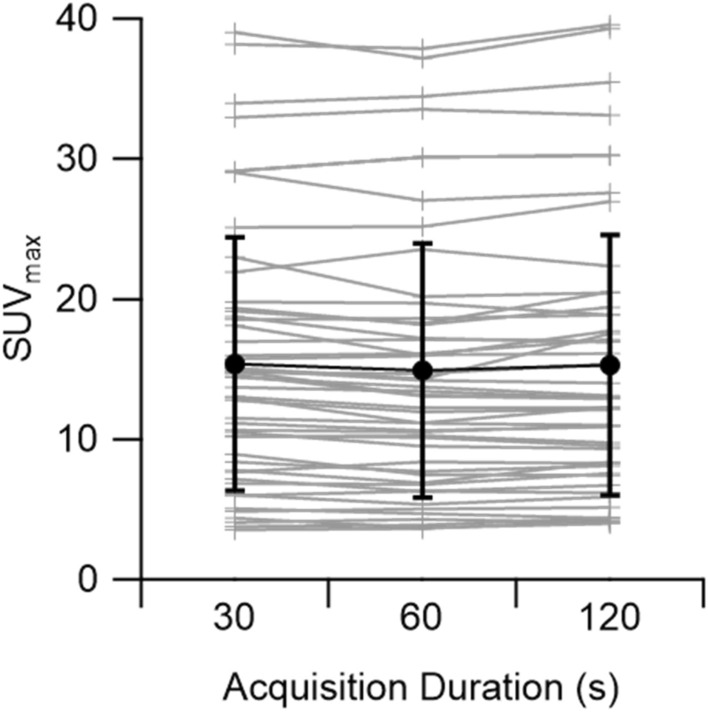
SUVmax for each lesion by acquisition duration (light grey). In bold (black) is the mean SUVmax ± standard deviation

We find a small variability in TBR (0.79%) when comparing the 30 s to the 2 min/bp reconstructions. Likewise, we find a smaller variability for the parameter SUVmean (0.35%) than for SUVmax (0.77%). In contrast to previous studies comparing the influence of shorter acquisition on SUVmax and SUVmean in a digital system [13] we find no statistically significant differences in SUVmax and TBR between the longer (2 min) and shorter (30 s) acquisitions. In Fig. 4 we show the SUVmax for the different acquisition durations. Shortening image acquisition durations broadens intensity distributions and should shift the supremum (of a set of values) found within a VOI towards higher values. Furthermore, low counts give rise to tail-heavy distributions in images reconstructed using OSEM and PSF-based algorithms [14], and the choice of reconstruction algorithm can have a significant effect on lesion quantification [13] Therefore, SUVmax, by definition the supremum, is expected to rise when lowering image count [14, 15]. However, when considering all SUVmax values found in our study, no clear differences can be seen between images for different list-mode acquisitions.

As described in the introduction, digital PET/CT represents a step forward in molecular imaging, with increasing numbers of publications confirming the favourable performance characteristics for such scans in comparison to previous-generation analogue systems [1, 16], including increased lesion detection and sensitivity [1, 3, 4, 13, 17]. Previous authors have reported head-to-head studies comparing digital and analogue systems, showing both improved image quality and upstaging of individuals [10], as well as improved recovery (as shown by higher SUV values) and lesion sharpness [3, 6]. Traditionally, shorter list-mode acquisitions have been associated with decreased lesion detection, as most recently shown for [^68^ Ga]Ga-PSMA-11 PET/CT by Rauscher et al. (using an analogue scanner) [18] with 1 min/bp reconstructions (equivalent to 1/3^rd^ of the standard) associated with reduced detection of up to 36% of lesions. We show here that applied dose reduction or reduction in acquisition time of 75% is feasible for the combination of [^18^F]-FDG and digital PET/CT without detriment with respect to lesion detection. Whereas extant procedural guidelines report protocols and standards for examinations for analogue scanners, the influence of digital PET/CT on list-mode acquisition length and applied dose has not been adequately addressed [7], increasing further the clinical importance of the topic.

Only a small number of publications considering these issues are available in the literature, and only few consider the impact of recent developments in PET technology. For example, Sonni et al. consider the influence of shorter acquisitions with a digital system on image quality (measured on a subjective 5-point scale), but do not report the influence on lesion detection [19]. In the context of combined PET/MRI scanners, multiple publications demonstrate the feasibility of reducing the [^18^F]-FDG dose using solid-state (silicon) photomultipliers [20, 21]. Van Sluis et al. consider the influence of shorter acquisitions in a digital PET/CT reporting both phantom [1] and clinical data, albeit in a small cohort of patients (*n* = 30) with no estimate of statistical power [13]. Although the authors of this latter study conclude that dose reduction is acceptable, they nevertheless report downstaging for one patient in this small cohort, albeit without any reported influence on therapeutic choice. Any putative benefit deriving from a small reduction in overall radiation exposure in an examination must be balanced against any potential clinical detriment arising from the misstaging of an established cancer—for example the harms caused by the missing of a contralateral lung nodule in a non-small cell lung cancer patient (NSCLC) far outweigh the benefit of a modest reduction in radiation exposure [22]. In contrast we find no detriment in a similarly small cohort, albeit with statistically defined endpoints and adequate statistical power to test our hypotheses. Larger studies must be performed to confirm this for all cancer types and in all patient groups. In their totality, we urge caution when interpreting these data. The equivalency of shorter acquisitions cannot be established with semi-quantiative lesion uptake or qualitative impression of image quality as the sole basis, and further work using clinically defined endpoints are required. The issue is therefore far from being exhaustively investigated, and prospective, randomized studies with larger cohorts are required to determine which patients can safely receive these shorter acquisitions.

Radiological protection concerns, however, are not the sole motivation of applied radioactivity dose reduction. Given the increasing utilisation of PET/CT for monitoring of therapeutics or treatment response, patients are increasingly re-referred for multiple restaging examinations, making accumulated radiation burden of potential relevance, particularly in neonates and infants. Given the limited supply of some radiopharmaceuticals, particulary those with short half-lives [23], we consider that reduction of dose/patient and faster examinations to be of considerable importance in increasing access to nuclear medicine examinations, where average post-referral waiting times for oncological PET/CT can be as high as 14 weeks in some jurisdications [24].

While longer acquisition times traditionally represent a balance between acquisition time and reduction in applied radiation dose, longer acquisitions can be associated with increased motion artefact, and point toward another key advantage in modern scanner design using proprietary end-expiratory PET acquisition for the reduction in respiratory motion artefact [25]. Likewise, while not the subject of this enquiry, the ability to increase numbers of patients examined through shorter-acquisition time is of considerable economic importance in an era of rapidly increasing utilisation of nuclear and molecular medicine imaging techniques.

We note some weaknesses with our study. Firstly, these initial experiences with a new scanner type are in *n* = 21 patients, although, in contrast to similar studies, a sample size calculation confirmed the adequacy of this cohort size to answer our hypothesis with adequate statistical power. Nevertheless, these findings must be considered preliminary and interpreted alongside the other similarly small cohorts hitherto published. We use clinically routine and vendor recommended reconstruction methods, EARL-accredited protocols may be better suited to quantitative PET studies in a multicentre setting [9]. However, the aim of our study was to show differences in vision detectability, and our chosen acquisition protocol was optimized for this in a clinical setting. We did not assess the influence of lesion size and note that in one case a small lesion was missed (Fig. 2). Previous studies report increased signal recovery from small structures in digital PET/CT systems [26], and further studies are needed to investigate the impact of shorter examinations or low dose protocols on small or low-uptake lesions. Reader bias was reduced using reviewers who had not seen the cases prior to the study. Furthermore, scans were read in randomized and blinded fashion. Few studies confirm the magnitude of any recall bias effect or confirm the optimum time between scan reading [27], although longer waiting periods may be beneficial. Instead, we highlight that scans were read starting with 30 s/bp (the images of lowest quality) and in randomized order with a pragmatic 48 h waiting period in between, limiting the impact of any recall bias inanycase. Other methods of reducing bias, such as using different readers for each list-mode reconstruction are not applicable to this study, where the magnitude of the interobserver agreement is likely larger than the magnitude of the absolute difference between scans (Krippendorf’s *α* = 0.99 in this study, inter-observer agreement in FDG PET *κ* = 0.69–0.79 [28]). Finally, institutional ethics board permission afforded anonymous and retrospective data acquisition: further studies, ideally of prospective design, are required to confirm the clinical non-inferiority of images acquired either with shorter-acquisition times, or by analogy lower radiopharmaceutical doses. Previous studies demonstrate that dose reduction is only feasible in non-obese individuals [20]. Our study included only two individuals with BMI > 30 kg/m^2^, and none with class II obesity (BMI > 35 kg/m^2^). Further studies in these population groups are required. Caution must be taken when generalising these results to other radiotracers, and further studies for non-oncological PET/CT (for example in PET for neurodegeneration) and using other tracers are required.

## Conclusion

We demonstrate that shorter acquisitions, or by analogy reduction in applied activity by as much as 75% is clinically feasible with no clear clinical detriment identified, using a digital PET/CT system. Agreement between these shorter acquisitions and the routine standard-of-care protocol is almost perfect (Krippendorf’s *α* = 0.99) both for lesion detection and tumour-to-background ratio. A small variability in SUVmax, SUVmean and TBR was seen (< 1%), with no clear relationship between shorter-acquisition times and variability. Our encouraging findings need to be confirmed by larger studies and different clinical settings.

## Ethical statement

The authors report no conflict of interest. A waiver was granted by the regional ethics committee for the retrospective, anonymous analysis of patient data (Req-2020–00678). The study was performed in accordance with the declaration of Helsinki. No funding was obtained.
